# Altered Interplay Among Large-Scale Brain Functional Networks Modulates Multi-Domain Anosognosia in Early Alzheimer’s Disease

**DOI:** 10.3389/fnagi.2021.781465

**Published:** 2022-02-03

**Authors:** Jose Manuel Valera-Bermejo, Matteo De Marco, Annalena Venneri

**Affiliations:** ^1^Department of Neuroscience, University of Sheffield, Sheffield, United Kingdom; ^2^Department of Life Sciences, Brunel University London, Uxbridge, United Kingdom

**Keywords:** anosognosia, large-scale networks, mild cognitive impairment, functional MRI, unawareness, resting-state

## Abstract

Decline in self-awareness is a prevalent symptom in Alzheimer’s disease (AD). Current data suggest that an early breakdown in the brain’s default mode network (DMN) is closely associated with the main symptomatic features in AD patients. In parallel, the integrity of the DMN has been shown to be heavily implicated in retained self-awareness abilities in healthy individuals and AD patients. However, the global contribution to awareness skills of other large-scale networks is still poorly understood. Resting-state functional magnetic resonance imaging (rs-fMRI) scans were acquired and pre-processed from 53 early-stage AD individuals. A group-level independent component analysis was run to isolate and reconstruct four intrinsic connectivity large-scale brain functional networks, namely left and right central executive fronto-parietal networks (FPN), salience network, and anterior and posterior DMN. Hypothesis-driven seed-based connectivity analyses were run to clarify the region-specific underpinnings of multi-domain anosognosia. Multiple regression models were run on large-scale network- and seed-based connectivity maps, including scores of memory, non-memory and total anosognosia obtained via the Measurement of Anosognosia Questionnaire. Memory anosognosia scores were associated with selective lower fronto-temporal connectivity and higher parieto-temporal connectivity. Non-memory anosognosia scores were associated with higher connectivity between the anterior DMN and the cerebellum, between the left medial prefrontal seeds and the contralateral prefrontal cortex, and between the left hippocampal seed and the left insula; lower connectivity was observed between the right prefrontal cortex and the right lingual seed. Lastly, total anosognosia scores were associated with large-scale network alterations, namely reduced left-FPN expression in the left posterior cingulate, reduced right-FPN expression in the left inferior lingual gyrus and adjacent inferior occipital cortex, and increased right-FPN expression in the right anterior cingulate. Seed-based analyses yielded significant connectivity differences only in the connectivity pattern associated with the left hippocampal seed by displaying lower intercommunication with the right prefrontal cortex, but higher connectivity with the left caudate nucleus. These findings support the hypothesis that alterations in functional connectivity of frontal lobe regions involved in executive-related mechanisms represent the neural correlates of domain-specific anosognosia in early AD. Up-regulated connectivity with subcortical structures appears to contribute to changes in the network dynamics interplay and fosters the appearance of anosognosia.

## Introduction

Anosognosia in Alzheimer’s disease (AD) is a prevalent symptom that can be defined as the inability of a patient to recognize decline in their own cognitive functioning (Hanseeuw et al., 2020). The clinical manifestations of anosognosia at an individual level affect efficiency in the various cognitive domains in a highly heterogeneous way (Starkstein, 2014). The onset of this symptom, however, tends to occur quite early on throughout the clinical timeline, either at the stage of mild cognitive impairment (MCI), or even before that, during the preclinical period when individuals who are still psychometrically normal but report subjective cognitive complaints might show poorer awareness of the extent of their decline than their informant would report (Cacciamani et al., 2017; Hanseeuw et al., 2020). Anosognosia might not manifest only as lack of awareness of a memory impairment but can also apply to other cognitive domains, such as unawareness of executive dysfunction, of socio-emotional deficits or of difficulties with daily life activities (Lacerda et al., 2021). This heterogeneity highlights the multidimensionality of this phenomenon and warrants the necessity for a multi-domain clinical assessment (Leicht et al., 2010; de Ruijter et al., 2020).

The use of resting-state fMRI (rs-fMRI) in AD holds great potential as an early diagnostic biomarker, as evidenced by studies that found disruptions of intrinsic large-scale network connectivity in the prodromal and mild AD stages (Sorg et al., 2007; Vemuri et al., 2012; Antoine et al., 2019). It is now well recognized that three of the brain main large-scale functional networks play a crucial role in efficient human cognition, namely the *default mode network* (DMN), the nodes of which are centered in the medial prefrontal cortex, posterior cingulate cortex/precuneus and medial temporal cortices, including the hippocampus (Raichle, 2015; Alves et al., 2019), the *salience networ*k, of which the anterior cingulate cortex and insula are the most representative hubs, and the *central executive fronto-parietal network*, with its computational regions found predominantly in the dorsolateral prefrontal and posterior parietal cortices (Bressler and Menon, 2010). Published evidence suggests that DMN down-regulation is significantly associated with cognitive decline during the early clinical stages of AD (Greicius et al., 2004; Badhwar et al., 2017; Grieder et al., 2018). At the same time, however, functional integrity of DMN hubs is implicated in self-awareness abilities, as evidenced in healthy individuals (Northoff et al., 2006; Davey et al., 2016). Consequently, AD patients with alterations affecting the DMN might have difficulties with self-awareness or may manifest anosognosia very early in the course of the disease.

To clarify the role of dysfunction in regions of the DMN when anosognosia is present in AD, partial insight is provided by fMRI studies that investigated hemodynamic activation in patients with AD during tasks tapping self-awareness abilities. These studies reported activation of fronto-parietal (Ruby et al., 2009), fronto-cingulate (Ries et al., 2007; Amanzio et al., 2011), and fronto-temporal (Zamboni et al., 2013) regions during tasks of self-reflection and perspective-changing, with an additional, yet limited, activation of posteromedial parietal regions (Amanzio et al., 2011). When resting-state activity within the DMN was studied in relation to anosognosia in this patient population, Berlingeri et al. (2015) found that anosognosia for memory impairment was associated with reduced connectivity of the DMN network, the left lateral temporal cortex, the hippocampus and the insula. Perrotin et al. (2015) found reduced connectivity between the medial temporal lobe and both orbitofrontal cortex and posterior cingulate. Vannini et al. (2017) showed reduced connectivity between the precuneus and the bilateral inferior parietal lobe, the left posterior cingulate and orbitofrontal cortex, and between the right hippocampus and the fusiform gyrus. Lastly, Mondragón et al. (2021) showed that stronger connectivity of the bilateral anterior cingulate cortex was associated with anosognosia in prodromal AD. Notably, regardless of the applied methodology, all task-based and rs-fMRI studies reported an involvement of the frontal lobe cortex and/or anterior cingulate cortex as the essential neural substrates modulating multi-dimensional awareness and self-appraisal abilities (Ries et al., 2007; Amanzio et al., 2011; Zamboni et al., 2013; Perrotin et al., 2015; Vannini et al., 2017; Antoine et al., 2019; Mondragón et al., 2021).

At the cognitive level, the most influential theoretical framework at the basis of disorders of awareness is the Cognitive Awareness Model (Agnew and Morris, 1998). This model posits that a “Mnemonic Comparator” based on executive resources plays a major role in sustaining awareness. It has, thus, been proposed that loss of functional integrity within fronto-temporo-parietal networks (including the DMN) may result in dysfunctional changes to the comparator system, and this, in turn, may lead to deficits in self-awareness abilities (Tagai et al., 2020). In this respect, an association between anosognosia and the integrity of the DMN has been reported by multiple studies (Zamboni and Wilcock, 2011; Antoine et al., 2019; Mondragon et al., 2019). Furthermore, the presence of anosognosia at the MCI stage has been found to predict hypometabolism in regions of the DMN and progression to dementia at a 2-year follow up (Therriault et al., 2018). The role that other, non-DMN large-scale networks play in the genesis and persistence of this symptom, however, is still poorly understood. Clarifying in more detail the changes to the overall neurofunctional circuitry (i.e., which networks are involved and in what direction the association is observed) that are associated with domain-specific anosognosia would provide valuable insights into the mechanisms fostering the presence of this symptom in AD.

Although the reviewed literature provides some evidence of the involvement of resting-state functional connectivity in the mechanisms of anosognosia in early AD, no study has yet investigated seed-based and data-driven (i.e., as informed by latent-variable models) pathways in a systematic way, including non-DMN networks, and by differentiating anosognosia for memory and non-memory impairments. Relying on the “Mnemonic Comparator” notion introduced as part of the Cognitive Awareness Model (Agnew and Morris, 1998), we hypothesized that connectivity alterations ascribable to fronto-limbic dysfunction would be associated with single-domain (i.e., “memory”/”non-memory”) and multi-domain (i.e., memory plus non-memory, or “total”) anosognosia in the early stage of AD. In addition, we also hypothesized that alterations to the DMN of adaptive and compensatory nature, such as increased abnormal network-to-network interplay, would be deployed (and, thus, would emerge as statistically significant) in the presence of anosognosia.

## Materials and Methods

### Experimental Design

This study followed a correlational design in which measures of domain-specific (memory and non-memory) and total anosognosia and statistical maps of brain activity extracted from fMRI images acquired in resting state were entered into linear regression models to explore the relationship between different brain network activities and patients’ levels of cognitive awareness.

### Participants

Fifty-three patients with cognitive impairment were included in this study. Recruitment of patients and administration of study procedures were carried out as an ancillary study of the EU-funded Virtual Physiological Human---DementiA Research Enabled by IT initiative^1^, a multicenter project coordinated by the Department of Neuroscience, University of Sheffield, United Kingdom. All eligible patients for the VPH-DARE initiative at our sites were also approached to take part in this ancillary study. All patients were recruited consecutively and all were approached if they also met the eligibility criterion for this additional study, i.e., having a reliable informant. Our intention was to cover the entire continuum of early AD clinical severity (i.e., from MCI to mild dementia) in order to maximize numerical variability of outcome variables (i.e., voxel-by-voxel indices of connectivity) as well as of the main predictors (i.e., the anosognosia scores). Of the total sample, *n* = 24 had a clinical diagnosis of mild AD, as per the National Institute of Aging diagnostic criteria typically implemented in clinical settings (McKhann et al., 2011) and *n* = 29 had a diagnosis of MCI due to AD (Albert et al., 2011) based on clinical, neuropsychological and structural neuroimaging biomarkers of neuronal injury (Albert et al., 2011, p. 8). Patient diagnoses were corroborated by regular clinical longitudinal follow ups that were carried out for at least 4 years after recruitment. In all cases included in this report the clinical course and structural imaging findings were supportive of an AD etiology. A series of exclusion criteria (applied at recruitment and, retrospectively, at each clinical follow up examination) was defined to rule out signs and symptoms suggestive of a condition incompatible with the objective of this study. This included: significant neurological conditions (e.g., history of acute or chronic cerebrovascular disease or transient ischemic attacks), history of epilepsy or presence of uncontrolled brain seizures, peripheral neuropathy, significant neuropsychiatric symptoms or evidence of radiological abnormalities (other than those seen as part of the typical AD profile); cardiovascular and gastroenterological conditions (e.g., sick-sinus syndrome or peptic ulcer), metabolic disorders (e.g., abnormal levels of vitamin B12, folates or thyroid stimulating hormone), major pharmacological interventions (e.g., treatment with psychotropic medication other than AD-related drugs, pharmacological interventions displaying important organic adverse effects or medications used in other research protocols) and presence of major disabilities significantly affecting daily life activities. Since the indices of anosognosia were obtained from patient-caregiver dyads, patients from the primary study without a reliable informant were not offered participation. A brief screening of caregivers’ neurological status was completed by a senior clinician to rule out neurological or psychological factors that could have prevented them from providing reliable information. This study received ethical approval by the Regional Ethics Committee of Yorkshire and Humber (Ref No: 12/YH/0474). In accordance with data-collection procedures approved by the European Union, ethical approval was reiterated by the relevant local ethics committees at recruitment sites. Written informed consent was obtained from all participants.

### Anosognosia Assessment

The *Measurement of Anosognosia Instrument* (Stewart et al., 2010) was used to quantify the patients’ level of (or lack of) awareness of difficulties in a set of daily life scenarios. This questionnaire consists of 15 dichotomical “yes/no” questions on cognitive deficits that may manifest during daily life routines and the responses are split into two functional domains: “memory” (9 items) and “non-memory” (including anosognosia for executive dysfunction and anosognosia for deficits in daily life activities; 6 items). All 15 questions must be answered independently by the patient and by the informant. As a result, two set of scores are obtained: that provided by the informant (construed as the “standard-of-truth” of the patient’s abilities) and that provided by the patient as a self-evaluative measure. Individual informant-based and patient-based responses were compared to quantify the number of discrepant answers (i.e., higher discrepancy scores indicate a higher degree of anosognosia). Discrepancy scores were used to quantify the presence of domain-specific anosognosia (“memory,” max score: 9; “non-memory,” max score: 6) and a “total” score that was also obtained by the sum of the memory and non-memory scores (max score: 15) (Migliorelli et al., 1995; Stewart et al., 2010).

### Functional Magnetic Resonance Imaging Acquisition and Pre-Processing

Brain MRI data were acquired following the protocol for multicenter harmonization for Philips MRI scanners defined by the VPH-DARE@IT consortium. Images were acquired on two Philips scanners. The following parameters were used for acquisition of resting state functional MRI (rs-fMRI) images on the 3 T Philips Ingenia scanner: 35 axial slices, reconstructed in-plane voxel dimensions = 1.8 × 1.8 mm^2^, slice thickness = 4.0 mm, 128 × 128 matrix size, 230 mm field of view, repetition time = 2.6 s, echo time = 35 ms, flip angle = 90°, number of temporal dynamics = 125. Acquisitions of rs-fMRI images on the Philips Achieva 1.5 T scanner had the following specifications: voxel size: 3.28 × 3.28 mm^2^, slice thickness = 6.00 mm, 64 × 64 matrix size, 230 mm field of view, repetition time = 2 s, echo time = 50 ms and flip angle = 90°, number of temporal dynamics = 240. As per protocol, 20 s of dummy acquisitions were set at the beginning of each sequence to enable the scanner to reach electromagnetic equilibrium. A T1-weighted image was also obtained for each participant as part of the MRI protocol.

Functional brain images were processed with Statistical Parametric Mapping (SPM) 12 software running in MATLAB R2014a (V.8.3) through a standardized fMRI data pre-processing pipeline that included slice-timing, realignment, normalization, temporal band-pass filtering (0.01–0.1 Hz) and a 6-mm full-width at half-maximum Gaussian kernel smoothing (Postema et al., 2019). In addition, total intracranial volumes were computed from the T1-weighted scans using the *get_totals* MATLAB script^2^ and summing the maps of gray matter, white matter and cerebrospinal fluid volumes.

### Independent Component Analysis

Pre-processed rs-fMRI images were analyzed with a group independent component analysis (ICA), to extract functional large-scale network connectivity maps. The GIFT toolbox (v1.3i)^3^ was used to this end. The Infomax optimization principle was applied and the number of components to be extracted set at 20, as a reliable number that typically separates human resting-state connectivity into its fundamental networks (Wang and Li, 2015). Individual independent component maps were reconstructed for each of the five main brain functional networks of interest and these were selected via visual inspection that was carried out independently by the three co-authors with 100% agreement. These five pathways were the anterior DMN (aDMN), the posterior DMN (pDMN), the left frontoparietal network (l-FPN), the right frontoparietal network (r-FPN), and the salience network. Large-scale network connectivity *beta*-score maps were then extracted for each participant for group-level inferential modeling.

### Seed-Signal Extraction and Seed-Based First Level Analyses

Region of interest (ROI) analyses were carried out on each individual patient’s rs-fMRI acquisition to define the association between the hemodynamic signal extracted from a series of seed regions and the signal throughout the entire brain. The resulting maps are typically interpreted as the functional connectivity of those seed regions. Cytoarchitectonically-defined seed regions were created using the WFU PickAtlas toolbox and AAL human brain atlas (Tzourio-Mazoyer et al., 2002; Maldjian et al., 2003). Informed by the Cognitive Awareness Model and based on previous neuroimaging research (Mondragon et al., 2019), the following 9 ROIs were chosen: anterior cingulate cortex, medial prefrontal cortex, medial orbitofrontal cortex, precuneus, and posterior cingulate cortex were selected for their established role in self-awareness (Northoff et al., 2006), while precentral gyrus, lingual gyrus, fusiform gyrus, and hippocampus were selected based on published evidence suggesting that these are part of the neural substrate associated with levels of anosognosia in the early stages of AD (Valera-Bermejo et al., 2020). Seed time-courses were extracted independently for the left and right hemispheres (18 seeds in total) with the SPM-based MarsBaR toolbox^4^ (Brett et al., 2002).

First-level analyses were devised in the form of general linear models with the seed time-course as predictor. The physiological signal from the global maps of white matter and cerebrospinal fluid was regressed out to minimize the impact of non-neural sources of variability. Twenty-four parameters related to head-motion i.e., the six rigid-body transformation regressors generated during realignment, their temporal derivatives, and all squared values were also regressed out (max. translational motion 3 mm and max. rotational motion 3°). This methodology also allowed for functional scans to address and minimize the impact of the differences in magnetic field strength as there is evidence that signal extent and intensity are not affected by this effect, but signal-to-noise ratio may variate depending on magnetic field-strength (Voss et al., 2006).

### Group-Level Data Modeling

Multiple regression analyses were carried out at group level to test the functional associations of domain-specific (i.e., memory and non-memory) and total anosognosia scores with large-scale network connectivity maps extracted through ICA and individual ROI maps obtained through seed-based first-level analysis. The cluster-forming threshold of significance was set at *p* = 0.005 uncorrected. Clusters were considered significant only if they survived a family-wise error (FWE)-corrected threshold of *p* < 0.05 (Whitwell, 2009). All statistical models were controlled for age, years of education, total intracranial volume, and normalized hippocampal volumes (Cardoso et al., 2013). The latter served to control for an index of neurodegeneration-informed disease severity. Peak regions were transposed from Montreal Neurological Institute coordinates to the Talairach space, to permit identification through the Talairach Daemon Client, version 2.4.3 (Lancaster et al., 2000).

## Results

### Demographics

Demographic and sample characteristics are reported in Table 1.

**TABLE 1:** Demographic characteristics of the sample.

Variable	Mean (*SD*)/*n* = 53	Median/*n* = 53	Range (min–max)
Age	71.68 (10.20)	77	48–89
Gender (%) (Male/Female)	27 (51%)/26 (49%)	−	−
Years of education	10.62 (4.05)	11	5–20
Mini-Mental State Examination	23.38 (3.77)	24	15–30
Total intracranial volume (mm^3^)	1422.63 (161.48)	1389.37	1131–1793
Memory anosognosia scores	1.34 (2.38)	2	−4–6
Non-memory anosognosia scores	1.04 (1.86)	1	−3–5
Total anosognosia scores	2.38 (3.75)	2	−6–10

### Large-Scale Network Results

**FIGURE 1:**
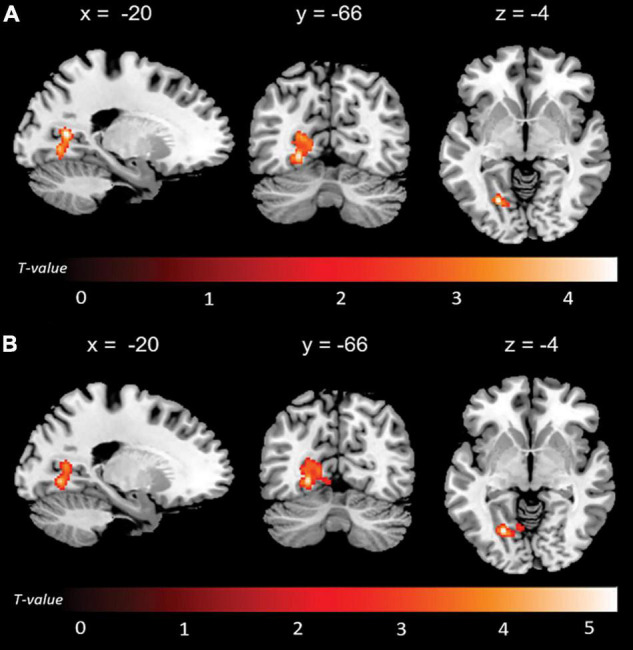
Negative correlations between functional connectivity of the left fronto-parietal network (l-FPN) and **(A)** memory and **(B)** total anosognosia.

**FIGURE 2:**
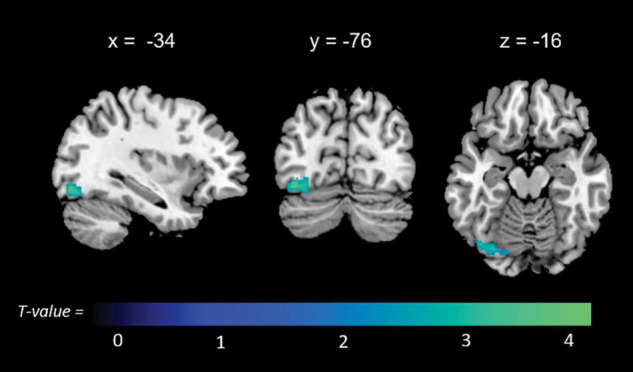
Positive correlations between functional connectivity of the right frontoparietal network (r-FPN) and total anosognosia.

**FIGURE 3:**
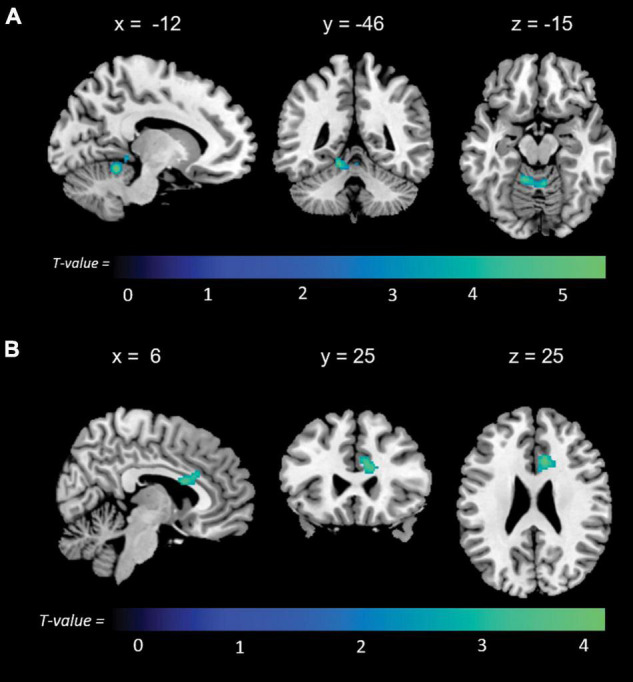
Positive correlations between functional connectivity of the anterior default mode network (aDMN) and **(A)** non-memory and **(B)** total anosognosia.

Significant associations emerged in some of the networks of interest (the extent of the clusters in voxels is indicated with *k*). Negative associations were found between expression of the l-FPN and both memory (*k* = 298, *p* = 0.02) and total anosognosia scores (*k* = 375, *p* = 0.006). These were in limbic-occipital regions, namely the left lingual gyrus and left posterior cingulate (Table 2 and Figure 1). Conversely, a significant positive association was found between expression of the r-FPN and total anosognosia (*k* = 264, *p* = 0.036). This was located in the left lingual gyrus and left inferior occipital gyrus (Table 2 and Figure 2). Along the same directional lines, positive associations were found between non-memory anosognosia scores and aDMN expression in the cerebellar culmen, bilaterally (*k* = 263, *p* = 0.038), and between total anosognosia and aDMN expression in the right anterior cingulate (*k* = 261, *p* = 0.039; Table 2 and Figure 3). No significant associations were found in the pDMN or salience network.

**TABLE 2:** Results emerging from large-scale brain functional networks obtained via independent component analysis.

Peak-based localization	HS	Cluster extent	T Score	MNI coordinates			FWE *P*-value
				*x*	*y*	*z*	
**l-FPN**							
**Memory anosognosia (−)**							
Posterior cingulate cortex (BA 30)	L	298	4.70	−20	−62	8	0.020
Lingual gyrus (BA 19)	L		4.59	−22	−68	−4	
Lingual gyrus (BA 18)	L		3.05	−16	−70	−12	
**Total anosognosia (−)**							
Lingual gyrus (BA 19)	L	375	5.22	−22	−68	−4	0.006
Posterior cingulate cortex (BA 30)	L		4.49	−24	−62	4	
Posterior cingulate cortex (BA 30)	L		3.75	−14	−66	4	

**r-FPN**							

**Total anosognosia (+)**							
Inferior occipital cortex (BA19)	L	264	4.89	−40	−74	−14	0.036
Inferior occipital cortex (BA19)	L		4.50	−36	−80	−10	
Lingual gyrus (BA 18)	L		3.36	−16	−84	−12	

**aDMN**							

**Non-memory anosognosia (+)**							
Cerebellum—culmen	L	263	5.21	−12	−48	−14	0.038
Cerebellum—culmen	R		4.94	4	−52	−14	
Cerebellum—culmen	L		3.49	−6	−36	−8	
**Total anosognosia (+)**							
Anterior cingulate cortex (BA 24)	R	261	4.35	10	18	20	0.039
Anterior cingulate cortex (BA 24)	R		3.70	16	28	16	

*(−), negative correlation; (+), positive correlation; aDMN, anterior Default Mode Network; BA, Brodmann Area; FWE, Family-Wise Error; HS, Hemispheric Side; l-FPN, left Frontoparietal Network; MNI, Montreal Neurological Institute; r-FPN, right Frontoparietal Network.*

### Seed-Based Analyses Results

**FIGURE 4:**
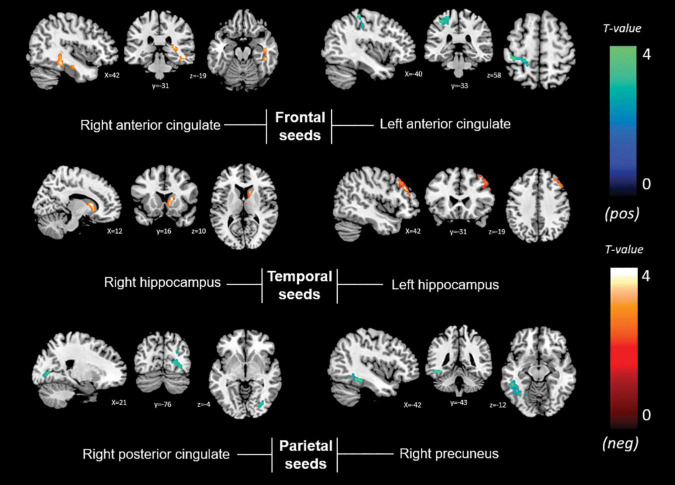
Positive (blue) and negative (red) correlations between brain functional connectivity of selected unilateral seed regions and memory anosognosia scores.

Memory anosognosia scores were negatively associated with: right anterior cingulate connectivity in a cluster extending from the right fusiform gyrus (BA 20) to the caudate (*k* = 383, *p* = 0.025); right hippocampal connectivity in the territory of the bilateral caudate and right thalamus (*k* = 394, *p* = 0.016); and left hippocampal connectivity in the right dorsolateral prefrontal cortex (*k* = 333, *p* = 0.040). Memory anosognosia scores were also positively associated with: right precuneal connectivity in the left fusiform and lingual gyri (*k* = 358, *p* = 0.028); right posterior cingulate connectivity in the right inferior occipital gyrus and cuneus (*k* = 407, *p* = 0.025); and left anterior cingulate connectivity in the precentral and postcentral gyri (*k* = 346, *p* = 0.029). All these results are summarized in Table 3 and Figure 4.

**TABLE 3:** Neural correlates of memory anosognosia emerging from functional-connectivity patterns obtained via seed-based analysis.

Peak-based localization	HS	Cluster extent	T score	MNI coordinates			FWE *P*-value
				*x*	*y*	*z*	
**Memory anosognosia**							
**Reduced connectivity**							
**Right anterior cingulate cortex seed**							
Caudate	R	383	3.96	30	−34	6	0.025
Fusiform gyrus (BA 20)	R		3.76	42	−36	−16	
Fusiform gyrus (BA 20)	R		3.54	48	−12	−20	
**Right hippocampus seed**							
Caudate	R	394	4.11	12	14	8	0.016
Thalamus	R		3.73	4	−4	4	
Caudate	R		3.57	14	18	−2	
**Left hippocampus seed**							
Middle frontal gyrus (BA 46)	R	333	4.78	46	34	28	0.040
Middle frontal gyrus (BA 9)	R		4.38	34	38	38	
Middle frontal gyrus (BA 9)	R		3.68	46	26	38	

**Increased connectivity**							

**Right precuneus seed**							
Fusiform gyrus (BA 37)	L	358	4.57	−48	−44	−14	0.028
Fusiform gyrus (BA 20)	L		4.42	−44	−36	−16	
Lingual gyrus	L		4.05	−26	−66	−4	
**Right posterior cingulate cortex seed**							
Posterior cingulate (BA 30)	R	407	4.15	30	−66	6	0.025
Inferior occipital gyrus (BA 19)	R		3.41	34	−74	−6	
Cuneus (BA 17)	R		3.39	22	−76	2	
**Left anterior cingulate cortex seed**							
Postcentral gyrus (BA 3)	L	346	4.52	−38	−32	58	0.029
Precentral gyrus (BA 4)	L		4.08	−30	−32	66	
Postcentral gyrus (BA 3)	L		3.85	−22	−36	60	

*BA, Brodmann Area; FWE, Family-Wise Error; HS, Hemispheric Side; L, Left; MNI, Montreal Neurological Institute; R, Right.*

**FIGURE 5:**
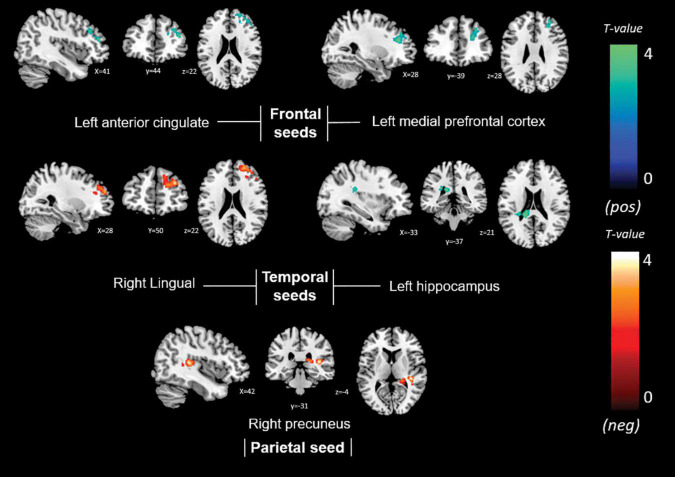
Positive (blue) and negative (red) correlations between brain functional connectivity of selected unilateral seed regions and non-memory anosognosia scores.

Non-memory anosognosia scores were negatively associated with: right lingual connectivity in the right dorsolateral prefrontal cortex (*k* = 590, *p* = 0.002); right precuneal connectivity in the right transverse temporal gyrus (BA 41) and in a caudate-thalamic area (*k* = 802, *p* = 0.001). Non-memory anosognosia scores were positively associated with: left anterior-cingulate connectivity in the right dorsolateral prefrontal cortex (*k* = 434, *p* = 0.012); left medioprefrontal connectivity in the right dorsolateral prefrontal cortex (*k* = 318, *p* = 0.048); and left hippocampal connectivity in a left caudate-insular cluster (*k* = 354, *p* = 0.031). All these findings are shown in Table 4 and Figure 5.

**TABLE 4:** Neural correlates of non-memory anosognosia emerging from functional-connectivity patterns obtained via seed-based analysis.

Peak-based localization	HS	Cluster extent	T score	MNI coordinates			FWE *P*-value
				*x*	*y*	*z*	
**Non-memory anosognosia**							
**Reduced connectivity**							
**Right lingual seed**							
Middle frontal gyrus (BA 9)	R	590	5.26	32	44	30	0.002
Middle frontal gyrus (BA 9)	R		4.50	24	54	22	
Superior frontal gyrus (BA 10)	R		3.38	12	54	32	
**Right precuneus seed**							
Transverse temporal gyrus (BA 41)	R	802	4.98	40	−32	10	0.001
Thalamus	R		3.99	24	−24	16	
Caudate	R		3.99	22	−36	10	

**Increased connectivity**							

**Left anterior cingulate cortex seed**							
Superior frontal gyrus (BA 10)	R	434	4.35	20	58	18	0.012
Middle frontal gyrus (BA 9)	R		3.88	40	46	20	
Middle frontal gyrus (BA 9)	R		3.63	28	40	32	
**Left medial prefrontal cortex seed**							
Middle frontal gyrus (BA 9)	R	318	4.59	26	36	22	0.048
Middle frontal gyrus (BA 9)	R		3.71	30	44	30	
Superior frontal gyrus (BA 9)	R		3.26	20	50	20	
**Left hippocampus seed**							
Caudate	L	354	3.88	−16	−38	20	0.031
Insula (BA 13)	L		3.25	−26	−36	24	

*BA, Brodmann Area; FWE, Family-Wise Error; HS, Hemispheric Side; L, Left; MNI, Montreal Neurological Institute; R, Right.*

**FIGURE 6:**
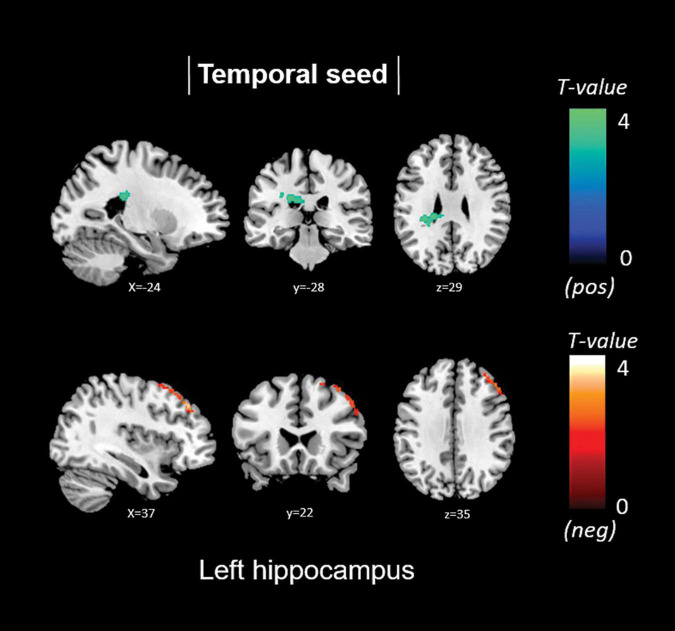
Positive (blue) and negative (red) correlations between brain functional connectivity of selected unilateral seed regions and total anosognosia scores.

Total anosognosia scores, finally, were negatively associated with left hippocampal connectivity in the right dorsolateral prefrontal cortex (*k* = 457, *p* = 0.009), and positively associated with the left caudate nucleus (*k* = 330, *p* = 0.042). These findings are reported in Table 5 and Figure 6.

**TABLE 5:** Neural correlates of total anosognosia emerging from functional-connectivity patterns obtained via seed-based analysis.

Peak-based localization	HS	Cluster extent	T score	MNI coordinates			FWE *P*-value
				*x*	*y*	*z*	
**Total anosognosia**							
**Reduced connectivity**							
**Left hippocampus seed**							
Middle frontal gyrus (BA 46)	R	457	4.91	48	32	28	0.009
Middle frontal gyrus (BA 9)	R		4.70	36	36	40	
Middle frontal gyrus (BA 6)	R		4.35	28	60	60	

**Increased connectivity**							

**Left hippocampus seed**							
Caudate	L	330	3.71	−16	−38	18	0.042
Caudate	L		3.57	−22	−30	26	

*BA, Brodmann Area; FWE, Family-Wise Error; HS, Hemispheric Side; L, Left; MNI, Montreal Neurological Institute; R, Right.*

## Discussion

The present study provides evidence of a specific combination of functional brain network dynamic changes associated with multi-domain anosognosia in the early stages of AD. A better understanding of the functional substrates of domain-specific anosognosia provides insight into how disconnection of structures at the basis of the Cognitive Awareness Model translates into different mechanisms, leading to impairment of self-awareness.

### Functional Connectivity Associated With Memory Anosognosia

Unawareness of memory deficits was linked to specific patterns of functional connectivity of large-scale neural networks and region-to-region pathways. Memory anosognosia scores were linked negatively to l-FPN functional connectivity within the left lingual gyrus and posterior cingulate (a DMN node). In addition, seed-based models showed a negative link with structures in the frontotemporal territory, i.e., the right fusiform gyrus (right anterior cingulate seed, aDMN associated structure), and the right dorsolateral prefrontal cortex (left hippocampal seed, DMN associated structure). It appears, therefore, that within the main human large-scale neural functional networks, there might be a possible inter-network down-regulation of FPN-DMN connectivity leading to memory anosognosia. A negative association was then found in the pathway between the right caudate and both right anterior cingulate and right hippocampal seeds. The involvement of temporal cortical structures in early AD patients presenting with higher memory anosognosia scores and functional connectivity alterations follows a pattern aligned with the pathophysiological progression of clinical AD. In this context, degeneration of the medial temporal lobe induces disruption of memory performance, but at the same time neuronal loss in these regions hampers functional interactions between crucial areas leading to reduced awareness of memory difficulties (Chavoix and Insausti, 2017). A histopathological study showed that AD patients with anosognosia had increased amyloid plaque density in the hippocampal pre-subiculum at *post-mortem* (Marshall et al., 2004). This finding, however, could have been partly driven by the severe pathological changes that typically affect the medial temporal lobe regions in the final stages of the disease. Pioneering studies that explored the neurofunctional substrates of memory anosognosia in AD through single-photon emission computerized tomography (Reed et al., 1993; Starkstein et al., 1995; Derouesne et al., 1999; Hanyu et al., 2008) or positron-emission tomography (Harwood et al., 2005; Jedidi et al., 2014) have highlighted right frontal cortical structures as the most consistent neural correlate. In a functional MRI study by Zamboni et al. (2013), AD patients showed decreased frontotemporal brain activation in relation to self-awareness. This evidence is aligned with our findings detecting an association with frontotemporal connectivity.

Additionally, memory anosognosia scores were also positively associated with connectivity between the right precuneus seed and the left lingual and fusiform gyrus and between the left anterior cingulate seed and left postcentral gyrus. Two recurring regions from our pattern of findings were the fusiform and lingual gyri. These structures have also been associated with anosognosia and reduced brain gray matter volumes in early AD (Guerrier et al., 2018; Valera-Bermejo et al., 2020). Functional alterations of the fusiform (Vannini et al., 2017) and lingual gyrus (Mitelpunkt et al., 2020) have been found to be associated with anosognosia in AD and might have strong links with regions within a broader network in charge of sustaining cognitive awareness. Increased frontal and parietal activity was found by Ruby et al. (2009) in a functional task-based fMRI study that showed higher activation in the prefrontal cortex of healthy controls in relation to AD patients when engaging in self-awareness tasks, while AD patients had higher activation of parietal cortices when engaging in tasks based on self-monitoring activities.

### Functional Connectivity Associated With Non-memory Anosognosia

Anosognosia for executive and everyday-life deficits was positively associated with aDMN connectivity in the cerebellum. Increased connectivity in the cerebellar cortex within the aDMN could contribute to improving the cross-talk with functional networks that are involved in higher cognitive functions, as it has been shown that structures in the cerebellum provide substantial contributions to cognition, e.g., executive functions (Nowrangi et al., 2014; Xu et al., 2020). Our findings also stress the relevance of subcortical structures in contributing to the DMN in the presence of self-awareness impairment, as evidenced by the role played by the cerebellum (Buckner et al., 2011; Alves et al., 2019).

Similarly, the more anosognosic, the stronger the functional connectivity pattern between left anterior brain seeds (anterior cingulate, a salience network node, and medioprefrontal cortex, a DMN node) and the right dorsolateral prefrontal cortex (a FPN node). A positive association was also found in the pattern of left hippocampal connectivity (DMN associated) with the left insular cortex (a salience network node) and left caudate. Findings in the insula show consistency with those in the literature centered on the neuroscience of self-awareness (Lou et al., 2017). One study that highlighted gray matter volumetric changes in left insular-hippocampal regions was carried out by Sánchez-Benavides et al. (2018) who found that individuals self-assessed as unaware of cognitive decline had larger gray matter insular volumes compared with individuals displaying subjective cognitive decline. Additionally, informant-related reports of cognitive changes (unrelated to self-awareness status) were associated with smaller gray matter volume in the left hippocampus. The latter study provides valuable insights about a link between insular-hippocampal alterations in psychometrically-normal older adults and abnormalities in cognitive awareness. While we found higher connectivity between the left hippocampus and insula in relation to non-memory anosognosia, Berlingeri et al. (2015) reported reduced connectivity between these same structures in dementia patients, but for memory anosognosia. These results indicate that insulo-mediotemporal regions disconnection might be pivotal for the onset of anosognosia, with the insula serving as a vicarious salience system in the presence of unawareness for executive and daily life functions, and a “mnemonic” hub relating instead to memory unawareness. Moreover, the insula has been found to be concomitantly active alongside the prefrontal cortex and anterior cingulate in tasks requiring self-referential processing, such as interoception, self-recognition, or socio-emotional awareness (Craig, 2009; Modinos et al., 2009). Higher connectivity with the executive fronto-parietal network in this structure has been found in association with higher scores on tasks involving awareness of others (tasks of social cognition/theory of mind) in patients with early stage AD (Valera-Bermejo et al., 2021). The insula, a core region of the salience network, might up-regulate its functional connectivity toward regions typically targeted by the AD etiopathological cascade to support network function, as a way to allocate additional neural resources in support of dysfunctional DMN pathways, in an attempt to minimize the loss of connectivity and, thus, sustain the circuits that are responsible for acknowledging the presence of illness. In summary, it seems that non-memory anosognosia might be modulated by the up-regulation of the executive frontoparietal and salience network in the presence of a weakened DMN, although any attempted compensatory effect is ultimately unsuccessful.

Additionally, significant negative associations were found within the map of right lingual connectivity in the right dorsolateral prefrontal cortex and within the map of right precuneal connectivity in right subcortical regions (caudate and thalamus). The findings of this study bring compelling evidence of an involvement of the right dorsolateral prefrontal cortex in modulating awareness of cognitive difficulties, with both higher connectivity observed in the left frontal lobe and lower connectivity observed in the right lingual cortex. The main nodes of the central-executive FPN are centered in the dorsolateral prefrontal cortex and posterior parietal cortex (Bressler and Menon, 2010). The dorsolateral prefrontal cortex has been associated with executive functions and working memory and a selective age-related vulnerability has been observed in the older population (MacPherson et al., 2002). Moreover, the right dorsolateral prefrontal cortex involvement in AD patients presenting with anosognosia has been evidenced in the current literature across multiple neuroimaging approaches (Reed et al., 1993; Starkstein et al., 1995; Ries et al., 2012). There is evidence that this region is part of an executive-control network that, in conjunction with the anterior cingulate cortex, coordinates behavior aimed at accomplishing life-related objectives (Cohen et al., 2000; Amanzio et al., 2011; Xu et al., 2020). In addition, the contribution of the dorsolateral prefrontal cortex to declarative memory has been established in AD (Kumar et al., 2017; Turriziani et al., 2019), as this structure is involved in working memory for manipulation and updating of conscious information that operates in close relation with a central executive system (Funahashi, 2017). Furthermore, the executive control network appears to rely on cerebro-cerebellar support in the presence of executive decline (Xu et al., 2020). Bi-directional dorsolateral prefrontal connectivity (i.e., a positive association found in contralateral frontal structures and a negative association found in ipsilateral temporal regions) might be at the basis of a role played by this region in sustaining adaptive cognitive control required by abilities such as attention (Gbadeyan et al., 2016).

### Functional Connectivity Associated With Total Anosognosia

Total anosognosia scores in this early AD sample were negatively associated with l-FPN expression in the left posterior cingulate, and seed-based analyses yielded significant negative connectivity associations between the left hippocampal seed and the right dorsolateral prefrontal cortex. These findings translate into changes in inter-network communication; indeed, the posterior cingulate and hippocampal regions are intrinsically associated with the DMN while the dorsolateral prefrontal cortex supports the executive fronto-parietal network (Bressler and Menon, 2010; Alves et al., 2019). Therefore, these findings provide evidence of reduced inter-communication between the DMN and the executive fronto-parietal networks.

Positive associations were found with r-FPN expression in the left inferior lingual gyrus and adjacent inferior occipital cortex, and between the aDMN expression in the right anterior cingulate (a salience network node). Therefore, total anosognosia might be the symptomatic expression of abnormally increased up-regulation of DMN-Salience inter-network connectivity in support of cognitive shifting of reduced self-related internal abilities to external salient stimuli. In addition, seed-based analyses revealed positive significant connectivity associations between the left hippocampal seed and the left caudate nucleus. The interplay between the fronto-parietal network and the lingual gyrus is in line with a hypothesis of frontally-mediated control of disorders of awareness. The lingual gyrus is a region essential for visual perception (Yang et al., 2015), but it also plays an executive role, as shown in a study that reported activation during a divergent thinking paradigm (Zhang et al., 2016).

A comprehensive overview of the results in the present study indicates that the anterior cingulate cortex has a strong involvement in the overall clinical manifestation of multi-domain anosognosia. Structural changes are commonly seen later than functional changes in the course of AD (Ewers et al., 2011), but there is, however, evidence that variability in gray matter of the anterior cingulate cortex is related to non-memory and total anosognosia in early AD (Valera-Bermejo et al., 2020). The present rs-fMRI findings extend the involvement of this structure to anosognosia for multiple cognitive domains. Regardless of the methodological approach, the anterior cingulate has been significantly implicated in modulating levels of awareness of symptoms in early AD (Hanyu et al., 2008; Amanzio et al., 2011; Zamboni et al., 2013; Guerrier et al., 2018; Mondragón et al., 2021). These results show consistency with the premise that midline anterior brain structures are essential for self-awareness and self-referential processing (Northoff et al., 2006; Lou et al., 2017). Structure and function of the anterior cingulate cortex could play a significant role alongside the resources deployed as part of the executive supporting system. This region could serve a major role in the executive comparator system described within the Cognitive Awareness Model (Agnew and Morris, 1998), in which the presence of synaptic dysfunction in anterior midline structures could generate a mismatch in the perceived reality and lead, therefore, to an event of cognitive unawareness. On this note, executive impairment, measured through a composite neuropsychological score, was shown to be associated with hypometabolism in the anterior cingulate in patients with a PET-informed diagnosis of prodromal AD, regardless of the extent of their amyloid burden (Yoon et al., 2019). Notably, the anterior cingulate has been found to be centrally involved in the manifestation of neuropsychiatric symptoms in AD (Boublay et al., 2016), where anosognosic symptoms are also listed as part of a spectrum of behavioral disorders that affects this clinical population (Tagai et al., 2020). In this context, the anterior cingulate cortex can be considered a core region that modulates behavior through reward, motivation, and initiation, i.e., the ability to commence a task (Devinsky et al., 1995). This could result in patients presenting with self-awareness deficits and displaying higher behavioral alterations when losing aspects of their sense of self.

### Subcortical Contributions to Anosognosia

Anosognosia scores were also associated with subcortical connectivity. Memory anosognosia scores were negatively associated with connectivity between the frontal and temporal cortex and the bilateral caudate and right thalamus. Non-memory anosognosia scores were also negatively associated with connectivity between the right precuneus and both right thalamus and caudate. Reduced functional connectivity between the medioprefrontal cortex and the caudate was also reported to be associated with the presence of memory anosognosia in early AD (Ries et al., 2012).

Increased connectivity was evidenced between the left hippocampus and left caudate. The latter pattern of results was also replicated when total anosognosia scores were analyzed. Subcortical contributions have been reported in dementia patients who overestimate their overall cognitive functions or during emotional control (Shany-Ur et al., 2014). This finding has been interpreted in the context of some subcortical regions being associated with the dopaminergic system that is involved in reward actions based on self-centered attention, resulting from life accomplishments (Shany-Ur et al., 2014). Dopaminergic activity has been proposed to enhance function in paralimbic structures involved in self-referential processing. Through their projections, these regions provide subcortical support to medial frontal cortical regions during conscious self-monitoring (Lou et al., 2017). Therefore, structural and functional alterations might result from adaptive, although ineffective, up-regulation of dopaminergic inputs in a systemic attempt to cope with symptoms related to unawareness.

**In summary**, taken together, the pattern of findings suggests that in the early stage of neurodegeneration there might be a rearrangement of activity within the main functional network dynamic interactions that normally support full awareness of cognitive function. Increases in activity can be interpreted as a surge in system effort that increases demands on neural resources without, however, succeeding in sustaining cognitive function. Increases in neural activity that do not result in successful performance have been reported before in MCI and early stage AD (e.g., Gardini et al., 2015) and interpreted as a progressive maladaptive reorganization of how neural resources are allocated and an early coping mechanism in response to progressive neural depletion. An alternative reading of the pattern of findings of this study would be to interpret them as a reflection of alterations in the equilibrium of activity between anticorrelated networks, in this instance the DMN and FPN, expression of their inherent functions in internal and external mediation, respectively (Fox et al., 2005). This pattern of alteration among these networks’ dynamic interactions was observed in association with all aspects of anosognosia in this study. Changes in inter-network activity expressed as increased connectivity between the DMN and the salience network, also found in this study in association with anosognosia, have been previously reported in mild probable AD dementia (Sarli et al., 2021). It might be suggested, therefore, that changes in activity in anticorrelated networks would alter the level of mutual inhibition exerted among their regions leading to a progressive loss of network integration and, in turn, to detectable cognitive and functional impairments, including alterations of awareness.

### Limitations

One of the main limitations of this study, and in general of the field of anosognosia research, is the choice of instrument to measure this symptom. Although, no method to detect anosognosia is recognized as the “gold-standard”, the analysis of discrepancy scores is currently the most accepted methodological approach (de Ruijter et al., 2020). Discrepancy scoring relies on the response given by both patient and informant. Caregiver burden may inadvertently shift the perception of the patient’s abilities into an over/under-estimation. To rule out this possibility, we chose to rely on a robust instrument that has undergone methodological validation. We acknowledge, however, that there are other ways to assess anosognosia, such as the discrepancy between subjective estimate of skills and actual objective performance on a task. Lastly, we cannot completely rule out the possibility that one of the two sub-scales may have had a larger impact on the total score than the other. The lack of large-scale findings within the salience network and pDMN connectivity might be due to insufficient sample size affecting statistical power to delineate the functional neural correlates of multi-domain anosognosia. This was a major reason why we chose to rely on the supportive evidence provided by seed-based models.

## Conclusion

Large-scale brain functional networks and seed-based findings showed negative fronto-temporal associations, while positive associations of parieto-temporal connectivity related with level of awareness of memory dysfunction in early AD. Conversely, awareness of symptoms within the non-memory (executive and daily-life) domain was positively linked to connectivity between the aDMN and cerebellum, between left hippocampus and left insula and between left medioprefrontal and right dorsolateral prefrontal cortex. Lastly, the total score reflective of the combined level of awareness of dysfunction in multiple cognitive and everyday-life domains showed a positive correlation with fronto-temporal pathways and with connectivity between the aDMN and the right anterior cingulate. The prefrontal cortex seems to be a critical mediator of single and multi-domain anosognosia in the early stages of AD.

**FIGURE 7:**
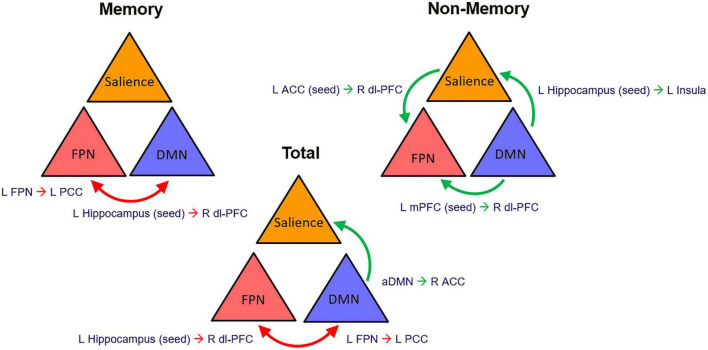
Graphical representation of the proposed network dynamic changes that define multi-domain anosognosia in the early stages of AD. ACC, anterior cingulate cortex; aDMN, anterior Default Mode Network; dl-PFC, dorsolateral prefrontal cortex; FPN, Fronto-Parietal Network; mPFC, medial prefrontal cortex; red arrows, decreased connectivity; green arrows, increased connectivity.

Contextualizing our findings based on the organization into nodes of the central large-scale brain functional networks, an overall pattern emerged of reduced interplay between the central executive FPN and the DMN influencing awareness for memory dysfunction as well as more globally when the memory and non-memory domain scores were summed together. On the other hand, increased intercommunication of the nodes within an executive system with both the DMN and salience network was found for non-memory anosognosia (Figure 7). This could translate into a reorganization of network dynamics in an attempt of the functional system to support its awareness abilities hampered by DMN dysfunction mechanisms. The dysfunction of a central executive comparator that influences levels of awareness could promote adaptive neural changes of increased network traffic to broader brain regions such as subcortical and supporting temporo-occipital territories (fusiform-lingual gyri) to sustain awareness in the early stages of AD degeneration. This neural coping attempts, however, do not appear to exert any behavioral benefit. These early changes in network dynamics provide insights on the evolution of deficits in awareness in AD where subtle alterations can be detected even at a preclinical stage (Cacciamani et al., 2017). Multi-domain assessment of awareness, therefore, could potentially provide early signs of worse disease outcome. In the presence of anosognosia, clinicians should suspect early brain functional network breakdowns that could potentially modulate negatively the phenotypical presentation of the disease and act as a potential trigger of comorbid neuropsychiatric manifestations. Awareness of the potential implications of this symptom should lead to better cognitive and therapeutic interventions.

## Data Availability Statement

The raw data supporting the conclusions of this article will be made available by the authors, without undue reservation.

## Ethics Statement

The studies involving human participants were reviewed and approved by the Regional Ethics Committee of Yorkshire and Humber (Ref No: 12/YH/0474). The patients/participants provided their written informed consent to participate in this study.

## Author Contributions

JMV-B contributed to data processing and data modeling, result interpretation, and writing of the initial draft of the manuscript. MDM contributed to data collection, result interpretation, and critical revision of the manuscript. AV contributed to study conception and funding, clinical assessment and diagnosis, and revising and finalizing of the manuscript. All authors contributed to the article and approved the submitted version.

## Conflict of Interest

The authors declare that the research was conducted in the absence of any commercial or financial relationships that could be construed as a potential conflict of interest.

## Publisher’s Note

All claims expressed in this article are solely those of the authors and do not necessarily represent those of their affiliated organizations, or those of the publisher, the editors and the reviewers. Any product that may be evaluated in this article, or claim that may be made by its manufacturer, is not guaranteed or endorsed by the publisher.

## Footnotes

1
https://cordis.europa.eu/project/id/601055

2
http://www0.cs.ucl.ac.uk/staff/g.ridgway/vbm/get_totals.m

3
mialab.mrn.org/software/gift

4
http://marsbar.sourceforge.net/
